# Antimicrobial Compounds in the Volatilome of Social Spider Communities

**DOI:** 10.3389/fmicb.2021.700693

**Published:** 2021-08-24

**Authors:** Alexander Lammers, Hans Zweers, Tobias Sandfeld, Trine Bilde, Paolina Garbeva, Andreas Schramm, Michael Lalk

**Affiliations:** ^1^Department of Cellular Biochemistry and Metabolomics, University of Greifswald, Greifswald, Germany; ^2^Department of Microbial Ecology, Netherlands Institute of Ecology (NIOO-KNAW), Wageningen, Netherlands; ^3^Section for Microbiology, Department of Biology, Aarhus University, Aarhus, Denmark; ^4^Section for Genetics, Ecology and Evolution, Department of Biology, Aarhus University, Aarhus, Denmark

**Keywords:** volatile organic compound, chemical ecology, antimicrobial, *Stegodyphus dumicola*, social arthropods

## Abstract

Social arthropods such as termites, ants, and bees are among others the most successful animal groups on earth. However, social arthropods face an elevated risk of infections due to the dense colony structure, which facilitates pathogen transmission. An interesting hypothesis is that social arthropods are protected by chemical compounds produced by the arthropods themselves, microbial symbionts, or plants they associate with. *Stegodyphus dumicola* is an African social spider species, inhabiting communal silk nests. Because of the complex three-dimensional structure of the spider nest antimicrobial volatile organic compounds (VOCs) are a promising protection against pathogens, because of their ability to diffuse through air-filled pores. We analyzed the volatilomes of *S. dumicola*, their nests, and capture webs in three locations in Namibia and assessed their antimicrobial potential. Volatilomes were collected using polydimethylsiloxane (PDMS) tubes and analyzed using GC/Q-TOF. We showed the presence of 199 VOCs and tentatively identified 53 VOCs. More than 40% of the tentatively identified VOCs are known for their antimicrobial activity. Here, six VOCs were confirmed by analyzing pure compounds namely acetophenone, 1,3-benzothiazole, 1-decanal, 2-decanone, 1-tetradecene, and docosane and for five of these compounds the antimicrobial activity were proven. The nest and web volatilomes had many VOCs in common, whereas the spider volatilomes were more differentiated. Clear differences were identified between the volatilomes from the different sampling sites which is likely justified by differences in the microbiomes of the spiders and nests, the plants, and the different climatic conditions. The results indicate the potential relevance of the volatilomes for the ecological success of *S. dumicola*.

## Introduction

Organisms use chemicals to exchange information, coordinate their behavior, or protect themselves against pathogens (Chen et al., 1998; Rowan, 2011; Schulz-Bohm et al., 2017). For example, social arthropods depend on chemical compounds, which are vital for communication and other functions mediating a high level of organization. They are able to inhabit extreme environments, use numerous resources, and finally often outcompete other arthropods (Wilson, 1987; Hölldobler and Wilson, 1990; Fisher et al., 2019). However, a fundamental problem of social arthropods is the elevated risk of acquiring and transmitting pathogens, as their dense colony associations increase the risk of infections and pathogen transmission (Bratburd et al., 2020). The risk of infections has led to a number of adaptations ranging from behaviors that reduce the risk of transmission (Müller and Schmid-Hempel, 1993) to the use of antimicrobial compounds. Antimicrobial compounds can be produced among others by the arthropod hosts themselves (Graystock and Hughes, 2011), symbiotic microorganisms (Musa Bandowe et al., 2009), or surrounding plants (Tariq et al., 2019).

Sociality has also evolved within the class of arachnids (Lubin and Bilde, 2007). *Stegodyphus dumicola* is a social spider species living in large groups in southern and central Africa (Aviles, 1997; Lubin and Bilde, 2007). These spiders build communal nests where reproduction takes place and which protects the spiders against heat/dehydration, UV-radiation, and predators (Supplementary Figure 1A; Seibt and Wickler, 1990; Henschel, 1998; Lubin and Bilde, 2007). The nest is surrounded by three-dimensional capture webs used for communal prey capture (Majer et al., 2018). The social lifestyle in spiders comes with elimination of pre-mating dispersal and therefore a strictly inbreeding mating system. Combined with frequent extinction and colonization events, this results in extremely low genetic diversity (Lubin and Bilde, 2007; Settepani et al., 2014, 2016, 2017). Homozygosity in genes within individuals, and low population genetic diversity in for example immune genes is likely to be associated with elevated vulnerability to infections. This substantiates the hypothesis that antimicrobial compounds play an important role in protecting the spider hosts against pathogens.

Due to the complex nest structure, including tightly woven silk structures with multiple narrow tunnels and chambers, volatile organic compounds (VOCs) have a possible importance in pathogen defense. VOCs are carbon-based compounds with molecular masses below 400 Da, high vapor pressures, low boiling points, and lipophilic moiety, properties that imply versatility of these compounds in all terrestrial ecosystems (Rowan, 2011; Schmidt et al., 2015b; Tyc et al., 2015). As opposed to soluble compounds, VOCs can diffuse through air-filled pores in complex ecosystems such as soil and hence do not depend on solvents (Schmidt et al., 2015a). That suggests that VOCs have the potential to reach all the internal surfaces of the spider nest and serve as inhibitors of pathogens from a distance. Antimicrobial VOCs are widely produced by microorganisms, plants, and animals: For example, bacteria isolated from the rhizosphere are known for producing VOCs with antimicrobial properties that can have protective functions for symbiotic plants (Tyc et al., 2015; Ossowicki et al., 2017). Plants use VOCs by themselves in various functions such as preventing microbial infections (Hammerbacher et al., 2019). In termites, volatile pheromones produced by queens, primarily used for communication, were shown to also have antifungal effects, indicating a role in pathogen defense (Matsuura and Matsunaga, 2015). Red fire ants and beetles are also known to use VOCs with antimicrobial properties (Gross et al., 2008; Wang et al., 2015). Here, we propose that VOCs may have a protective antimicrobial function in the nest system of the social spider *S. dumicola*.

The aim of this study is to describe the volatilomes of the spider *S. dumicola* and its nest and web, respectively, and to assess its potential antimicrobial effects. We hypothesized the presence of antimicrobial VOCs in the volatilomes of *S. dumicola*, its nest, and catching web. Next, we investigated whether the spider, the nest, and the capture web emit different VOC blends. Finally, we investigated volatilomes collected at different geographical locations to assess differences between the VOC blends. For this, we analyzed and compared the volatilomes from spider nests, catching webs, and the spiders themselves at three sampling sites in a north-south gradient in Namibia. Furthermore, we tested a selection of identified VOCs as pure compounds for their antimicrobial activity against microbial pathogens of spiders and humans.

## Materials and Methods

### Sampling Sites

The volatilome samples were taken at three different sampling sites in Namibia between 8th–26th February, 2019 (Supplementary Figure 2). The locations were close to the Etosha National Park (“Otavi”; S19.47, E17.19), the capital (“Windhoek”; S22.57, E17.21), and a small town (“Stampriet”; S23.74, E18.19). Otavi and Stampriet are at an altitude of ∼1,300 m and Windhoek of ∼2,000 m. At each sampling site five nests were analyzed and defined as biological replicates. The maximal distance between the used spider nests were 150 m in Otavi, 300 m in Windhoek, and 1,200 m in Stampriet (Supplementary Figure 2 inserts). Otavi is a humid sampling site in the North, Windhoek is mountainous, and Stampriet is because of its closeness to the Kalahari Desert on average the warmest region.

In Otavi the nests were located in plants of the species *Combretum imberbe*, *Acacia mellifera*, *Ziziphus mucronata*, and *Grewia flava*; in Windhoek in *Acacia hereroensis*, *Acacia mellifera*, and *Acacia hebeclada*; in Stampriet in *Acacia nebrownii*. We empirically observed the highest density of plants in Otavi and lowest in Stampriet.

The humidity and temperature data during the volatilome trapping were measured using iButton^®^ logger every 300 s (Type DS1923, Maxim Integrated, San Jose, California, United States). The temperature and humidity data inside the nests during the VOC samplings revealed the nests in Otavi as warmest and less humid and the nests in Windhoek as less warm and most humid (Supplementary Figure 3).

### Volatilome Trapping

Polydimethylsiloxane (PDMS) tubes (internal diameter 1 mm, external diameter 1.8 mm, Carl Roth, Karlsruhe, Germany) were cut into 5 mm pieces and threaded on needles. The tubes were fully covered with acetonitrile/methanol (4/1, v/v) and incubated for 3 h at room temperature. Subsequently they were dried under N_2_ flow (5 l/min) and heated up to 210°C for 1.5 h under He flow (5 l/min). Glass vials for storing the tubes until usage and needles for fixation at the spider nests and webs were cleaned similarly.

The PDMS tubes were used to trap volatile organic compounds (VOCs) from the spider nests, catching webs, and isolated living spiders. For trapping VOCs from the nests, five tubes were fixed with needles at the nest surfaces (Supplementary Figure 1B). For trapping VOCs from the catching webs, five PDMS tubes were threaded on needles to increase the surface and stuck to the catching webs in an approximately 30 cm diameter around the nests. PDMS tubes in sterile, open Petri dishes in 1 m distance to the nests were used as controls. The nests and webs were irritated as little as possible before, during, and after VOC trapping to stress the spiders as little as possible. No spiders or material were removed before trapping. VOCs from spiders were trapped by keeping five spiders from each nest together with five PDMS tubes in a sterile Petri dish. The controls for spider VOCs were closed Petri dishes with five PDMS tubes. All PDMS tubes were left for 30 min and immediately stored in closed glass vials at −20°C until VOC analysis. One nest (together with its catching web and spiders) was defined as one replicate. Per sampling site 3–5 replicates were taken.

### GC/Q-TOF Analysis

The VOCs were released from the PDMS tubes using an automated thermodesorption unit (Unity TD-100, Markes International, Llantrisant, United Kingdom) at 280°C for 8 min with a He flow of 50 ml/min. The VOCs got cold trapped at −10°C on a Tenax trap (Markes International, Llantrisant, United Kingdom) and released at 300°C within 10 min. A split ratio of 1/4 was used. The VOCs were transferred (195°C transfer line) to the Agilent 7890B GC (Agilent Technologies, Inc., Santa Clara, CA, United States) with an DB-5 ms ultra inert column (30 m length, 0.25 mm internal diameter, 0.25 μm film thickness, 122–5,532, Agilent Technologies, Inc., Santa Clara, CA, United States) and a run time of 35.6 min. The temperature program was set to 39°C for 1 min followed by heating up to 315°C with 10°C/min and holding for 7 min. The MS (280°C transfer line) was performed with Agilent 7200AB Q-TOF at 70 eV in electron ionization mode with a source temperature of 230°C. Mass spectra were recorded in full-scan-mode (m/z 30–400, 4 scans/s, 2 GHz Extended Dynamic Range).

For calibration of the retention index 1 μl alkane standard solution of C_8_–C_20_ (40 mg/l in Hexane; 04070-5ML; Merck, Darmstadt, Germany) was spiked on an empty Tenax trap and measured as described above. The presence of acetophenone, 2-decanone, 1-decanal, 1,3-benzothiazole, 1-tetradecene, and docosane (1 μg/ml in MeOH; all purchased by Merck, Darmstadt, Germany) in the volatilomes was confirmed by measuring pure standard compounds on the same way.

### GC/Q-TOF Data Processing

For GC/Q-TOF data processing the raw data were exported as content definition file (CDF) and imported into MZmine (Version 2.20;^©^ Copyright 2015; Pluskal et al., 2010). Mass detection, chromatogram building, deconvolution (local minimum algorithm), and peak alignment (RANSAC, random sample consensus) were performed using MZmine (for detailed parameters are provided in Supplementary Table 1). The peak lists were exported as comma-separated values (CSV) files. The files were uploaded to MetaboAnalyst (Version 4.0, Xia Lab, Montreal, Canada; Xia and Wishart, 2011), filtered (interquartile range), transformed (log transformation), and scaled (auto) before statistical tests were performed. Statistically significant differences between the samples and controls were identified by analysis of variance (ANOVA) followed by Fisher’s least significant difference (LSD). Furthermore, a mass feature must be found in at least 4 of 5 or 3 of 4 of the biological replicates to get valued as such.

Compound identification was performed using AMDIS 2.72 (National Institute of Standards and Technology, United States) based on retention index comparison and mass spectrum comparison to three libraries providing more than 1.4 million spectra namely NIST 2014 V2.20 (National Institute of Standards and Technology, Gaithersburg, Maryland, United States), Wiley 7th edition spectral libraries (Wiley, Hoboken, New Jersey, United States), and an internal library of NIOO-KNAW (Netherlands Institute of Ecology, Wageningen, The Netherlands). The retention index tolerance was ± 10 and the minimum mass spectrum match was 600 ‰. If a mass feature complied both criteria, a visual comparison of the mass spectra was performed, and designated as “tentatively identified.” Additionally, the retention indices and mass spectra of the “identified” compounds were compared to pure standard compounds measured in the same GC/Q-TOF system. Unknown mass features were assumed as different ones when the retention indices differed by > 6.

### Antimicrobial Test of Pure VOCs

Five VOCs identified using pure standard compounds, namely acetophenone, 2-decanone, 1-decanal, 1,3-benzothiazole, and 1-tetradecene, were tested as pure compounds for their antimicrobial activity. The compounds were selected based on literature indicating their high antimicrobial activity (Supplementary Table 2). We used *Bacillus thuringiensis* (DSM 2046), *Staphylococcus aureus* (DSM 799), *Escherichia coli* (DSM 787), and *Candida albicans* (DSM 10697) as test strains to cover all, a suggested spider pathogen, Gram positive and negative bacteria, as well as a yeast. The latter three strains are common human pathogens and were chosen as model organisms. All strains were bought at the German Collection of Microorganisms and Cell Cultures (DSMZ, Braunschweig, Germany). Before use the strains were pre-cultured overnight at 37°C on Mueller Hinton Agar II (2.0 g/l beef heart infusion, 17.5 g/l acid casein hydrolysate, 1.5 g/l starch, 17.0 g/l agar; Becton Dickinson, Franklin Lakes, New Jersey, United States).

The test strains were diluted to an OD_600_ of 0.1 in Mueller Hinton Broth II (17.5 g/l casein acid hydrolysate, 3 g/l beef extract, 1.5 g/l starch; Sigma-Aldrich, St. Louis, Missouri, United States). 10 ml of the prepared cell solution was transferred into a 100 ml-Erlenmeyer flask and test compounds, were added at a final concentration of 30 mM. The flasks were sealed immediately with aluminum foil and wrapping film and incubated (37°C, 150 rpm). After 24 h the OD_600_ was measured and compared to the negative control without added compounds (*t*-test, *p* ≤ 0.05). Three biological replicates for each test strain in combination with each test compound were performed.

Additionally, an agar diffusion test was performed based on the protocol of Hudzicki (2009). The test strains were diluted to an OD_600_ of 0.125 in NaCl (0.9%) and spread on Mueller Hinton II agar plates (Becton Dickinson, Fraklin Lakes, New Jersey, United States) using cotton swabs. Five microliters of the pure compounds were pipetted on empty cotton disks (6 mm diameter) and placed on the plates before sealing with Parafilm^®^. Empty cotton disks were used as negative controls. For positive controls cotton disks with gentamicin (Sensi-Disk^TM^, 10 U, Becton Dickinson, Franklin Lakes, New Jersey, United States) were used for bacterial strains and amphotericin B (ROTI^®^ Antibiotic Disks, 100 U, Carl Roth, Karlsruhe, Germany) for the yeast. After incubation (24 h, 37°C) the zones of inhibition (ZOI) were measured.

### Identification of Differences Between Volatilomes

To compare the volatilomes between the nests, catching webs, and spiders, and between the sampling sites, partial least squares discriminant analysis (PLS-DA) plots and Euler plots were made. PLS-DA plots were based on all mass features (including relative intensities) and made using MetaboAnalyst (Version 4.0, Xia Lab, Montreal, Canada; Xia and Wishart, 2011). Euler plots were made based on all compounds (Supplementary Table 2) VOCs using RStudio (RStudio, Inc., Version 1.2.5033).

## Results

### Antimicrobial VOCs in the *Stegodyphus dumicola* Volatilome

The analyses of the volatilomes of all sampling sites resulted in the tentative identification of 53 compounds ranging from C_4_ up to C_24_ (Table 1 and Supplementary Table 2). Most of the identified VOCs were pure hydrocarbons (41%) or contained oxygen (51%). Eight percent of compounds contained nitrogen, sulfur, or halogens. The VOCs belonged to various chemical classes. Most common were alkanes, carboxylic acids, alcohols, benzenes, ketones, alkenes, aldehydes, and terpenoids. One hundred forty-six compounds could not be tentatively identified by mass spectra comparison with databases and are therefore listed as unknown (Supplementary Table 2). Antimicrobial activity was assigned to 21 of the 53 tentatively identified VOCs (Table 1) based on published data for the pure compounds or mixtures such as essential oils containing the compound (references in Supplementary Table 2). Most of those compounds are known for both antibacterial and antifungal activities. By analyzing pure standard compounds, we confirmed the presence of acetophenone, 2-decanone, 1-decanal, 1,3-benzothiazole, 1-tetradecene, and docosane (Supplementary Figure 4).

**TABLE 1:** List of identified compounds in the nest (N), web (W), and spider (S) volatilomes of the different sampling sites Otavi, Windhoek, and Stampriet with known antimicrobial activity.

					Otavi			Windhoek			Stampriet		
Compound	Class	Molecular Formular	Antimicrobial Activity	Compound Origin	N	W	S	N	W	S	N	W	S
1-Heptanal	Aldehyde	C_7_H_14_O	b, f	p						×			
2-Ethylhexanol	Alcohol	C_8_H_18_O	b, f	p, f				×	×		×	×	
Acetophenone	Ketone	C_8_H_8_O	b	?	×			×					
1-Non-anal	Aldehyde	C_9_H_18_O	b, f	p	×						×		
Levomenthol	Alcohol	C_10_H_20_O	b, f	p		×							
2-Decanone	Ketone	C_10_H_20_O	b, f	p, b				×			×		
Dodecane	Alkane	C_12_H_26_	b, f	p, b							×		
1-Decanal	Aldehyde	C_10_H_20_O	b, f	p	×	×							
1,3-Benzothiazole	Benzothiazole	C_7_H_5_NS	b, f	p	×	×		×					
1-Undecanol	Alcohol	C_11_H_24_O	b	?								×	
1-Dodecene	Alkene	C_12_H_24_	b	p							×		
1-Tridecene	Alkene	C_13_H_26_	b	p, a	×	×		×	×		×		
2-Ethyl-3-hydroxyhexyl 2-methylpropanoate	Carboxylic Acid	C_12_H_24_O_3_	b, f	p	×								
1-Tetradecene	Alkene	C_14_H_28_	b, f	p, f			×						
1-Dodecanal	Aldehyde	C_12_H_24_O	b, f	p							×		
Nerylacetone	Ketone	C_13_H_22_O	b	p	×	×						×	
1-Dodecanol	Alcohol	C_12_H_26_O	b	p		×	×	×			×		
Pentadecane	Alkane	C_15_H_32_	b, f	a, i							×	×	
Myristic acid	Carboxylic Acid	C_14_H_28_O_2_	b, f	p	×						×		
Heneicosane	Alkane	C_21_H_44_	b	p							×	×	
Docosane	Alkane	C_22_H_46_	b, f	p							×	×	

*The antimicrobial activity of the VOCs was shown against bacteria (b) and/or fungi (f). The VOCs originated from plants (p),insects (i), algae (a), bacteria (b), and/or fungi (f). Antimicrobial activities and origins are based on literature data (references in Supplementary Table 2). Detection of VOCs in the samples is indicated by “ × ”.*

### Effect of Antimicrobial VOCs on Pathogens

**FIGURE 1:**
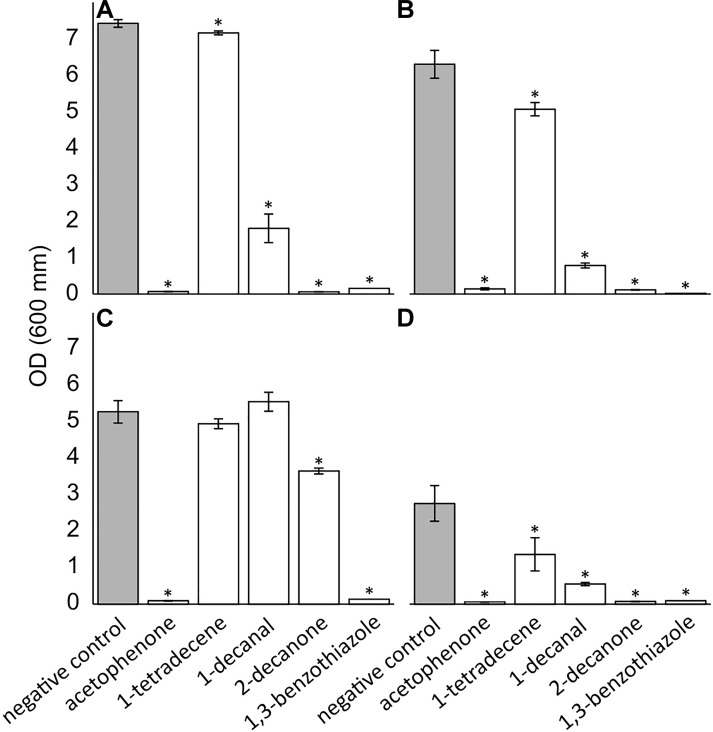
Antimicrobial effect of pure VOCs identified in the volatilomes of *Stegodyphus dumicola*. The compounds were tested at 30 mM against *Bacillus thuringiensis* **(A)**, *Staphylococcus aureus* **(B)**, *Escherichia coli* **(C)**, and *Candida albicans* **(D)**. The error bars show the standard deviation. The * indicate significant decreases in OD_600_ after 24 h incubation; *t*-test, *p* ≤ 0.05; *n* = 3.

A selection of pure compounds found exclusively in the nest volatilome (acetophenone and 2-decanone), the nest and web volatilome (1-decanal and 1,3-benzothiazole) and in the spider volatilome (1-tetratdecene) were tested for their antimicrobial activities against a spectrum of microbial pathogens. All five tested compounds displayed antimicrobial activity (Figure 1). Acetophenone, 2-decanone, and 1,3-benzothiaziole significantly inhibited all four tested pathogens. 1-Tetradecene and 1-decanal significantly inhibited only three pathogens, namely *B. thuringiensis*, *S. aureus*, and *C. albicans* but not *E. coli*. 1-Decanal even increased the growth of *E. coli* albeit not significantly. The spider pathogen *B. thuringiensis* was significantly inhibited by all five compounds. Additionally, we tested the antimicrobial activity of the compounds using an agar diffusion test resulting in similar inhibitions (Supplementary Figure 5).

### Comparison of Nest, Web, and Spider Volatilomes

**FIGURE 2:**
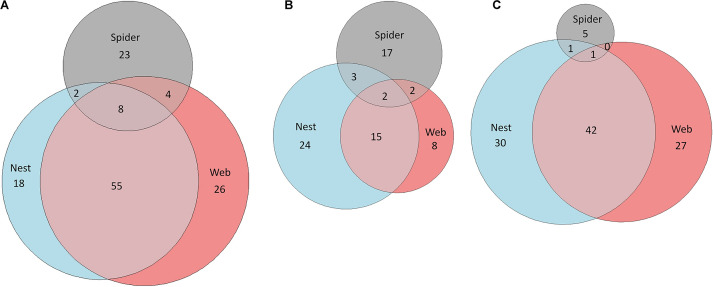
Comparison of the nest, web, and spider volatilomes using Euler diagrams. The diagrams are based on all identified and unknown VOCs from Otavi **(A)**, Windhoek **(B)**, and Stampriet **(C)**. The areas are proportional to the number of VOCs.

At all sampling sites, more VOCs were detected in the nest and web samples as compared to the spider samples (Figure 2 and Supplementary Figure 6). The nest and web volatilomes from the site Otavi shared many common VOCs (63 in total, Figure 2A). Only eight unknown VOCs were commonly found in the nest, web, and spider volatilomes. In Windhoek we found nearly twice the number of VOCs in the nest compared to the web and spider volatilomes (Figure 2B). Two unknown VOCs were common and detected in the nest, web, and spider volatilomes. In Stampriet, similar to Otavi, the nest and web shared a high number of VOCs (Figure 2C). Only one compound was found in the combined volatilome of nest, web, and spider and identified as oxymethylencampher.

### Comparison of Volatilomes From Different Climatic Regions

**FIGURE 3:**
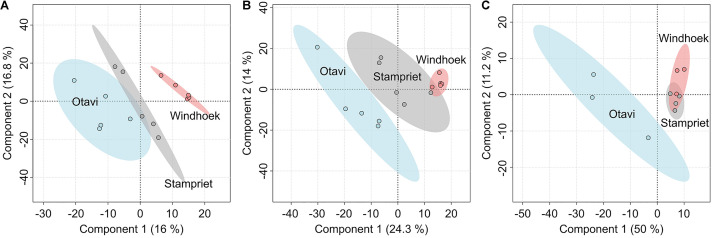
Comparison of the volatilomes of the sampling sites Otavi, Windhoek, and Stampriet using partial least square discriminant analyses (PLS-DA). The diagrams are based on all identified and unknown VOCs of the nests **(A)**, webs **(B)**, and spiders **(C)**. The semi-transparent colored areas show the 95% confidence regions.

Analyzing the volatilomes of the three sampling sites using PLS-DA indicated that the nest volatilomes from the three sampling sites clearly differed from each other, with the largest differences being observed between Otavi and Windhoek (Figure 3). The confidence regions of Otavi and Stampriet showed only small overlays (Figure 3A). The web volatilomes separated in a similar way with a small overlayed area between Stampriet and Windhoek (Figure 3B). The confidence regions of the spider VOCs of Stampriet overlayed in large parts with those of Windhoek, whereas the Otavi volatilome was clearly separated from the other regions (Figure 3C).

**FIGURE 4:**
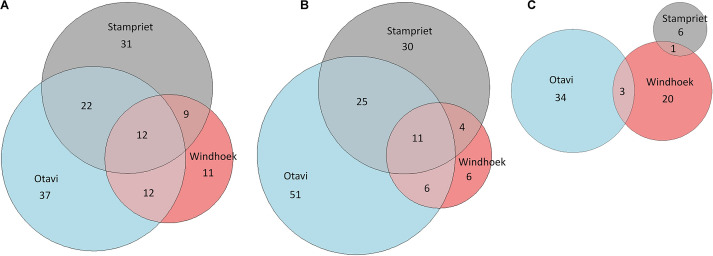
Comparison of the volatilomes of the sampling sites Otavi, Windhoek, and Stampriet using Euler diagrams. The diagrams are based on all identified and unknown VOCs of the nests **(A)**, webs **(B)**, and spiders **(C)**. The areas are proportional to the number of VOCs.

The Euler-plots based on all identified and unknown VOCs indicated that the Otavi volatilomes show the highest number of VOCs in the nest, and web volatilomes, followed by Stampriet, and Windhoek (Figure 4 and Supplementary Figure 6). The spider volatilomes contained the highest number of VOCs in Otavi and the lowest in Stampriet. The nest volatilomes of the three sampling sites shared 12 common VOCs, and the web 11 common VOCs. The spider volatilomes did not contain common VOCs among sites. 1-Methoxy-2-propanol and 1-tridecene were detected in all nests. 2,6,11-Trimethyldodecane was detected in all web volatilomes. The other shared VOCs remain unknown.

## Discussion

The results of our study revealed the presence of numerous VOCs with antimicrobial function in the *S. dumicola* volatilomes. Overall, around 40% of the identified VOCs in the volatilomes of *S. dumicola* exhibit antimicrobial activity based on literature (references in Supplementary Table 2). In the present study, several pure VOCs, namely acetophenone, 1-tetradecene, 1-decanal, 2-decanone, and 1,3-benzothiazole, displayed antimicrobial activity against a range of spider and human pathogens. The suggested spider pathogen *B. thuringiensis* (Keiser et al., 2016) and the human pathogens *S. aureus, E. coli*, and *C. albicans* (Peterson, 2009; Canet et al., 2018) were significantly inhibited by most of the tested VOCs, indicating a possible protection of *S. dumicola* against bacteria (Gram-positive and -negative) and yeasts. Our results are in line with several other studies that tested the antimicrobial activity of these compounds as pure compounds or extracts with concentrations between 0.55–43.8% (Palic et al., 2002; Shi et al., 2010; Tayung et al., 2011; Li et al., 2012; Liu et al., 2012; Guleria et al., 2013; Kazemi and Sharifi, 2017). For example, acetophenone and 2-decanone as pure compounds showed both antibacterial and -fungal activity (Rajabi et al., 2005; Sivakumar et al., 2008; Zheng et al., 2013; Jayakumar et al., 2020). The appearance of other antimicrobial VOCs was shown in other arthropod systems, for example in termites (Chen et al., 1998; Matsuura and Matsunaga, 2015), beetles (Gross et al., 2008), and ants (Wang et al., 2015). We found between 7 and 14 antimicrobial VOCs at each sampling site indicating likewise a high potential of the *S. dumicola* volatilomes in pathogen protection.

**FIGURE 5:**
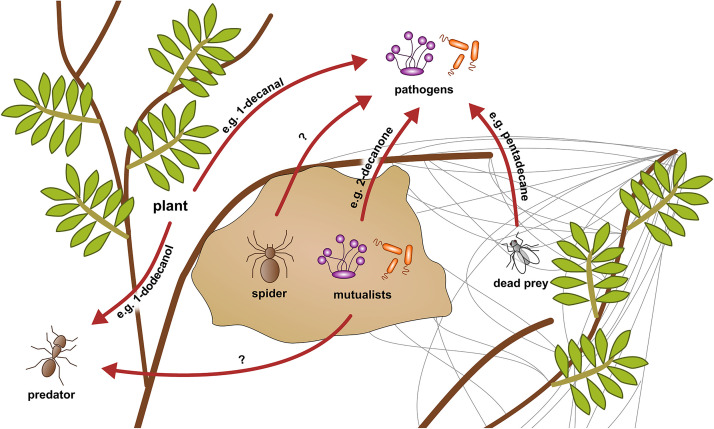
Conceptual scheme of hypothetical VOC interactions in a social spider nest system. VOCs can be originated from the spiders themselves, their microbiome, dead prey, or the plants and inhibit pathogens and predators (red arrows). The example VOCs were found in the analyzed volatilomes of the present study. Their origin and influence are assumed based on literature.

The origin of the VOCs in *S. dumicola*’s volatilome appears to be diverse—in principle, the spiders themselves, symbiotic microorganisms, prey (and their microbiota), the plants in which the nests are located, and even passing or hostile animals could influence the volatilomes (Figure 5). Most of the VOCs with known antimicrobial activity identified in this study were previously reported from essential oils of plants, but some also from fungi, bacteria, algae, or even insects (Table 1). The lowest number of VOCs was found in the volatilomes of isolated spiders, whereas relatively more VOCs were detected in the nest and web volatilomes. Furthermore, the spider volatilomes only shared a small number of VOCs with the nest and web volatilomes, whereas the nest and web shared many common VOCs. Particularly the nest may provide a source for a diverse community of microbes as plant material and exoskeletons are incorporated in the silk structure providing potential substrates for microbes. As the capture web is much more exposed to wind compared with the nest, we expected less VOCs in the web volatilomes than in the nest volatilomes, however, this was only the case in Windhoek.

A likely origin of VOCs is the *S. dumicola* nest microbiome. Although diverse and differing in composition and relative abundance from nest to nest, four bacterial genera (*Curtobacterium, Modestobacter, Sphingomonas, and Massilia*) and four fungal genera (*Aureobasidium, Didymella, Alternaria, and Ascochyta*) were found in all investigated *S. dumicola* nests and thus form a core nest microbiome (Nazipi et al., 2021). Currently, we cannot link specific VOCs to specific genera. However, the microbiome is the source of both soluble (Currie et al., 1999; Chouvenc et al., 2013; Mendes et al., 2013) and volatile (Musa Bandowe et al., 2009) antimicrobial compounds in other social arthropods, and some of the VOCs we identified in the spider nests are produced by microorganisms. For example, 2-decanone was found in the volatilomes of three *Bacillus* species and showed antifungal activity (Yuan et al., 2012; Zheng et al., 2013; Che et al., 2017; Jayakumar et al., 2020). Furthermore, 1-tetradecene was antimicrobial against numerous bacteria and fungi (Tayung et al., 2011). Another probable origin of the VOCs might be the arthropods themselves. A study identified pentadecane, an antimicrobial compound (Ozdemir et al., 2004; Hussain et al., 2017), in extracts of the head and gaster from argentine ants (Cavill and Houghton, 1974), and we identified the same compound in spider nests and webs in Stampriet. Pentadecane might have originated from dead prey or even hostile ants, even when we didn’t observe the latter during field work. The spiders themselves may be a source of the volatilome. For example, it was shown that the cuticular profile of *Stegodyphus lineatus* contains several linear and branched alkanes (Grinsted et al., 2011) and myristic acid plays a role in sexual signaling in *Tegenaria* spp. (Trabalon et al., 1997). Most of the antimicrobial VOCs found in the volatilomes were identified in essential oils of plants, which contain volatile and non-volatile compounds, suggesting that the plants in which the spiders build their nests may influence the nest volatilome. For example, triterpenes extracted from *Combretum imberbe* and *Acacia mellifera* showed antibacterial activity (Angeh et al., 2007; Mutai et al., 2009a). The majority of the plant species in which the spider nests were located in, are known for their antibacterial, antifungal, and/or antiviral activity, even when detailed studies on the chemical compositions are lacking (Masoko et al., 2007, 2010; Peloewetse et al., 2008; Mutai et al., 2009b; Arbab et al., 2015; Lamola et al., 2017; Shikwambana and Mahlo, 2020). All in all, we found several VOCs in the nest, web, and spider volatilomes. The highest diversity was found in the nest and web samples even though the spiders are likely exposed to a mixture of all VOCs present in the spider nest ecosystem. It is likely that the volatilomes are produced communally by bacteria, fungi, plants, and the spiders, respectively, even though we have only little hints to concrete origins yet (Figure 5).

The PLS-DA analyses show clear differences between the three sampling sites for each of the analyzed volatilomes (nest, web, spider). The Euler plots support that finding, as the majority of the VOCs (approx. 94%) were only found at one or two locations. There are various potential factors influencing the volatilomes. The nest microbiomes of *S. dumicola* were shown to differ significantly between different sampling sites, even though there is a core microbiome on genus level (Nazipi et al., 2021). A study investigating the spider microbiome of *S. dumicola* (endo- and exosymbionts) showed significant differences even between spiders from different nests from the same sampling site (Busck et al., 2020). Therefore, differences of the nest and spider microbiomes between the different geographic sites might contribute to the differences between the volatilomes found in the present study. The microbiome itself can be shaped by external factors like temperature, humidity, prey, soil, and plant species (Reese and Dunn, 2018). We found clear differences in temperature, humidity, and plant species between the sampling sites, which also goes in line with the differences between the volatilomes. The quantity and quality of the prey is also likely influenced by the location and climate (Majer et al., 2013, 2018). Furthermore, the plants themselves are a potential VOC source (Hammerbacher et al., 2019) and differed clearly between the sampling sites with a much higher plant density in Otavi compared to Windhoek and Stampriet (own observation). It was also shown that the production of essential oils by plants is decreased with increasing altitude (Haider et al., 2009; El-Jalel et al., 2018) which is in line with our results. Thus, the lowest number of VOCs was found at the highest altitude (Windhoek). In contrast to the spider microbiome, it was shown that *S. dumicola* shows little genetic differences between sampling sites (Settepani et al., 2017), which makes the spiders themselves very unlikely as an influencing factor for the differences between locations. Even though most of the VOCs were not shared between the sampling sites, some VOCs were present at all sites which suggests a site-independent “core volatilome” of *S. dumicola*. 12 VOCs were found in the nest volatilomes of all sampling sites and 11 VOCs in all web volatilomes. Two tentatively identified VOCs, namely 1-methoxy-2-propanol and 1-tridecene, were found in all nest volatilomes and 2,6,11-trimethyldodecane and 1-tridecene in all web volatilomes. None of these compounds is known from other social insect or spider systems but 1-tridecene showed antibacterial activity (Kumar et al., 2011; Satmi and Hossain, 2016) and 1-dodecanol, a compound we found at all sampling sites in the nests or webs, is well known for its insecticidal (Tabanca et al., 2014) and antibacterial activity (Togashi et al., 2007; Vairappan et al., 2012). It is conceivable that this compound may protect *S. dumicola* against hostile insects like ants or wasps, but also against bacterial pathogens (Figure 5).

In conclusion, we found distinct differences between the sampling sites that can be explained with differences in the microbiomes, plant quality and quantity, prey quality, and physical parameters like temperature, humidity, and altitude. We lack information about the *in situ* concentrations of the VOCs, because it is technically impossible to combine minimal invasive, untargeted, and *in situ* volatilome analysis with quantitative conclusions. In soil systems it is known, that aggregates creates micro-“incubators” influencing microbial life (Rillig et al., 2017). Similar phenomena may be possible in the spider nest ecosystem influencing the growth of certain microorganisms and the accumulation of antimicrobial VOCs. Nonetheless, we found hints for a core volatilome that may be important for the ecological success of *S. dumicola*. Overall, our study revealed that the volatilomes of *S. dumicola* contain numerous VOCs with antimicrobial potential that might play a key role in their pathogen defense. Apart from the tentatively identified VOCs the majority of ∼75% remains unknown. Therefore the *S. dumicola* system might contain more antimicrobial VOCs with the potential to protect the spiders and reveal novel classes of antimicrobial compounds. Next to antimicrobial protection VOCs can also play an important role in communication which should be addressed in further studies (Weisskopf et al., 2021).

## Data Availability Statement

The original contributions presented in the study are included in the article/Supplementary Material, further inquiries can be directed to the corresponding author/s.

## Author Contributions

AL, AS, HZ, ML, PG, and TS designed the study. AL performed the volatilome trapping, GC/Q-TOF data analysis, and the antimicrobial testing, and wrote the manuscript with support from AS, PG, and TB. TS assisted in planning and realization of the fieldwork. HZ performed the GC/Q-TOF analysis. All authors reviewed the manuscript and approved the submitted version.

## Conflict of Interest

The authors declare that the research was conducted in the absence of any commercial or financial relationships that could be construed as a potential conflict of interest.

## Publisher’s Note

All claims expressed in this article are solely those of the authors and do not necessarily represent those of their affiliated organizations, or those of the publisher, the editors and the reviewers. Any product that may be evaluated in this article, or claim that may be made by its manufacturer, is not guaranteed or endorsed by the publisher.
