# Supplementary material for: Antimicrobial Compounds in the Volatilome of Social Spider Communities
Source: Front Microbiol. 2021 Aug 24;12:700693. doi: 10.3389/fmicb.2021.700693 (PMC8422909; doi:10.3389/fmicb.2021.700693)
Supplement: Supplementary file 1 [file Data_Sheet_1.docx]

# Data Availability Statement

The dataset for this study can be found in the zenodo.org database under the number 5034289. DOI: 10.5281/zenodo.5034289

# Supplementary Material


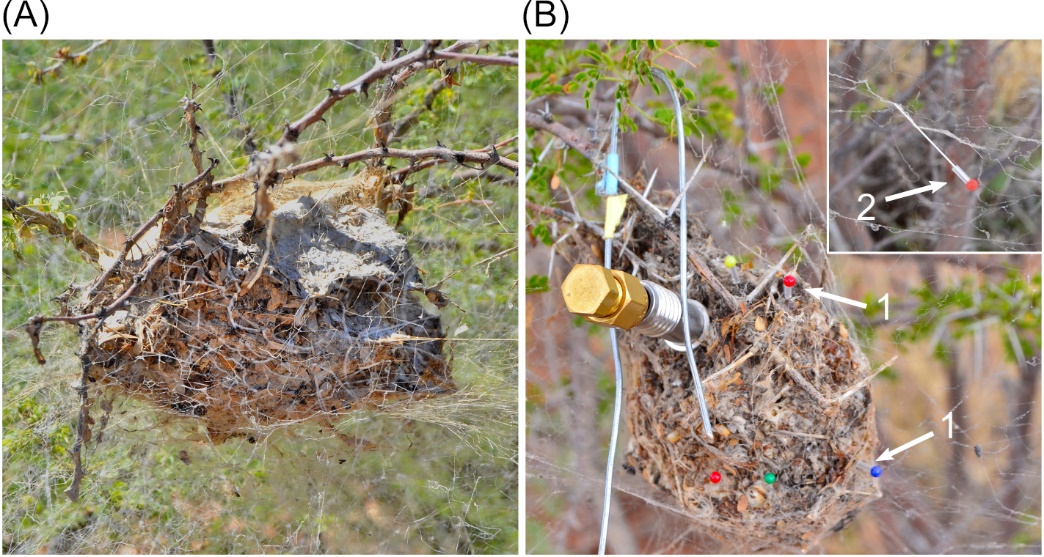


**Figure S 1 | Photographs of the nest of *Stegodyphus dumicola* and experimental design of VOC trapping*.*** (A) A nest with a diameter of approximately 20 cm. (B) Polydimethylsiloxane (PDMS) tubes at the nest (1) and the web (2) were used to trap VOCs. The PDMS tubes were combined with needles for better fixation.


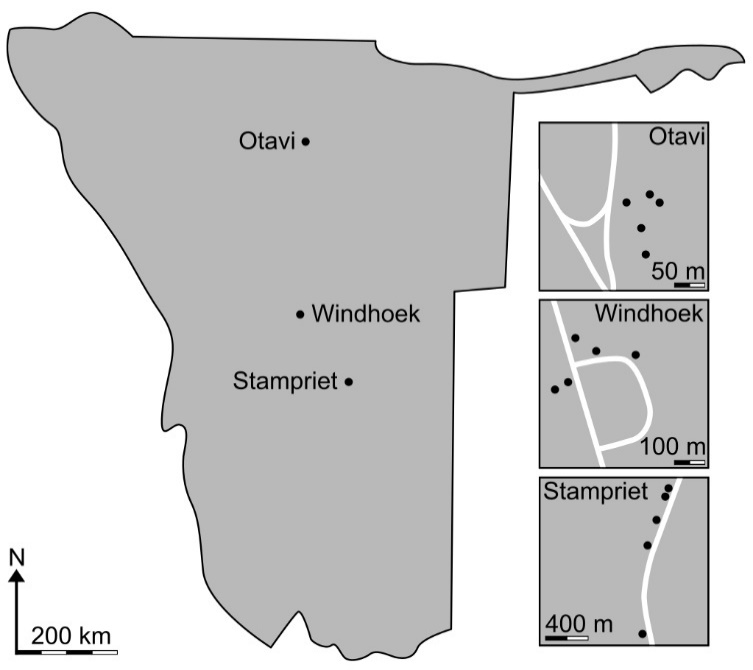


**Figure S 2 | Map of Namibia showing the sampling sites.** The inserted maps show the distributions of the spider nests at the sampling sites Otavi (S19.47, E17.19), Windhoek (S22.57, E17.21), Stampriet (S23.74, E18.19).


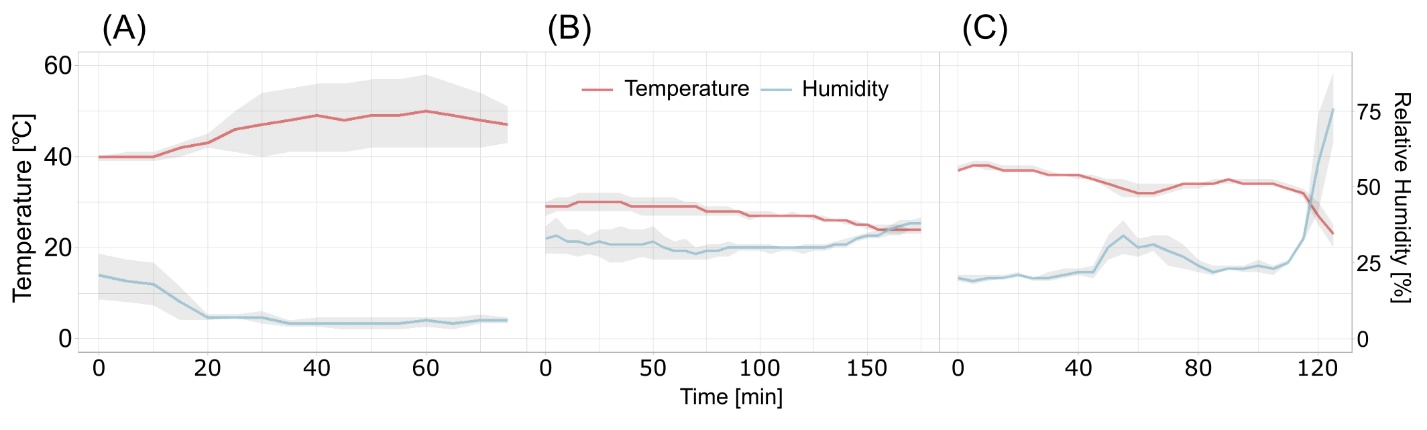


**Figure S 3 | Temperature and humidity data of the spider nests during VOC trapping in Otavi (A), Windhoek (B), and Stampriet (C).** The grey shadows indicate the maximum span of the parameter. Otavi/Stampriet *n* = 3; Windhoek *n* = 4

**
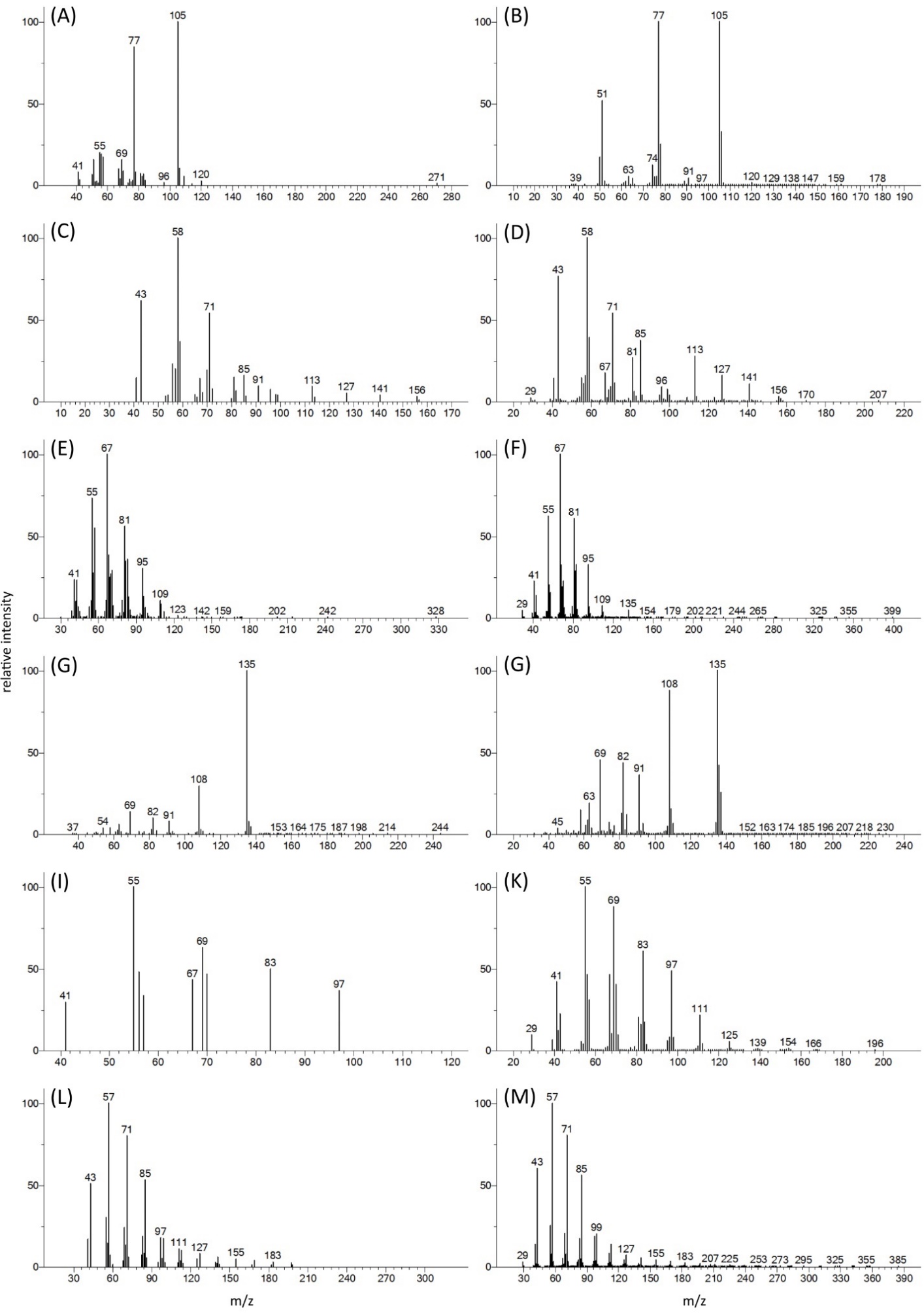
Figure S 4 | Mass spectra of the identified compounds (*in situ* sample/pure analytical standard) acetophenone (A/B), 2-decanone (C/D), 1-decanal (E/F), 1,3-benzothiazole (G/H), 1-tetradecene (I/K), and docosane (L/M).**

**
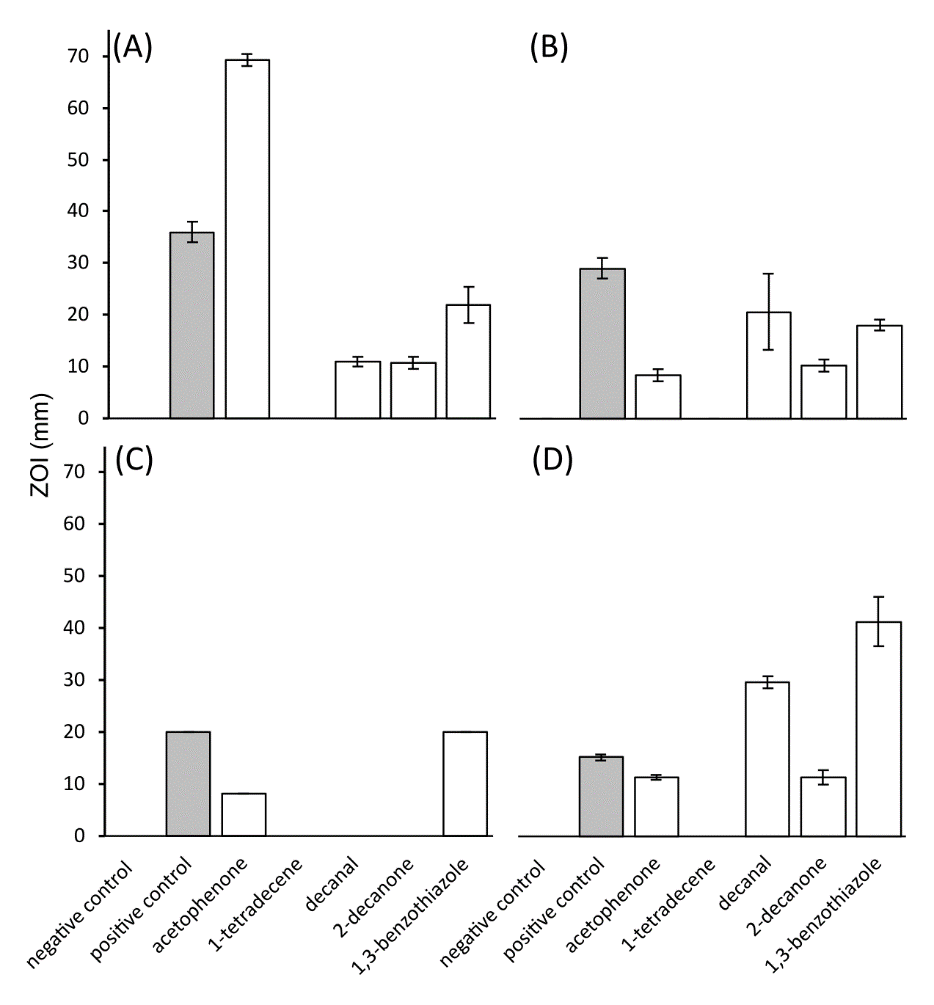
Figure S 5 | Antimicrobial effect of pure VOCs identified in the volatilomes of *Stegodyphus dumicola*.** The compounds were tested using the agar diffusion test against *Bacillus thuringiensis* (A), *Staphylococcus aureus* (B), *Escherichia coli* (C), and *Candida albicans* (D). The error bars show the standard deviation. *n* = 3.


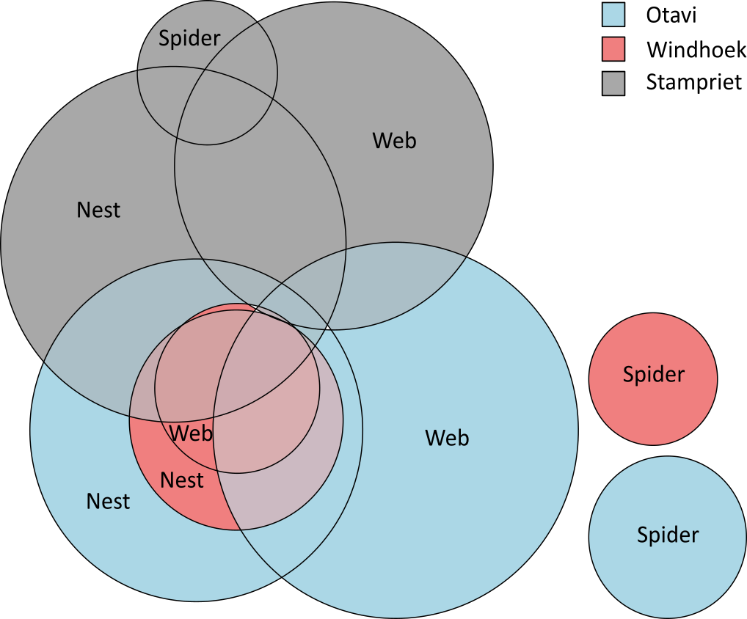


**Figure S 6 | Combined comparison between the sampling sites (Otavi, Windhoek, Stampriet) and sampling types (nest, web, spider) using a Euler diagram.** The diagram is based on all identified and unknown VOCs. The areas are proportional to the number of VOCs.

**Table S 1 | Parameters used for data processing** **using MZmine.** RT – retention time, RANSAC - random sample consensus

|  |  |  |
| --- | --- | --- |
| Process Step | Parameter [Unit] | Value |
| Mass Detection | Noise Level | 1.00E+02 |
| Chromatogram Building | Minimum Time Span [min] | 0.001 |
|  | Minimum Height | 1.00E+03 |
|  | m/z Tolerance [mz / ppm] | 1 / 5 |
| Chromatogram Deconvolution (Local Minimum Search) | Chromatographic Threshold [%] | 35 |
|  | Search Minimum in RT Time [min] | 0.03 |
|  | Minimum Relative High [%] | 10 |
|  | Minimum Absolute Height | 1.00E+03 |
|  | Minimum Ratio of Peak Top/Edge | 2 |
|  | Peak Duration Range [min] | 0-0.5 |
| Peak Aligment (RANSAC) | m/z Tolerance [mz / ppm] | 0.001 / 1 |
|  | RT Tolerance [min] | 0.1 |
|  | RT Tolerance After Correction [min] | 0.05 |
|  | RANSAC Iterations | 10,000 |
|  | Minimum Number of Points [%] | 70 |
|  | Threshold Value | 1 |
|  |  |  |

**Table S 2 | Complete list of tentatively identified and unknown compounds found in the volatilomes of *Stegodyphus dumicola* at the sampling sites Otavi, Windhoek, and Stampriet in the nests (N), webs (W), and spiders (S).** The presence of a compound in a sample (indicated by “×”) was assumed when a significant mass feature compared to the control in 4/5 or 3/4 of the biological replicates were found (ANOVA and Fisher’s LSD). The compounds were tentatively identified by comparison of the mass spectra and retention indices with databases. The antimicrobial properties of the compounds were shown in the references. RT – retention time, RI – retention index, match – similarity of the measured mass spectrum with the mass spectrum of the database

|  |  |  |  |  |  |  | | |  | | |  | | |  |
| --- | --- | --- | --- | --- | --- | --- | --- | --- | --- | --- | --- | --- | --- | --- | --- |
|  |  |  |  |  |  | Otavi | | | Windhoek | | | Stampriet | | |  |
| Compound | Class | Molecular  Formular | RT [min] | RI | Match [‰] | N | W | S | N | W | S | N | W | S | References |
| unknown |  |  | 2.2652 | 565.6 |  |  |  |  |  |  |  |  |  | × |  |
| unknown |  |  | 2.7692 | 610.6 |  |  |  |  |  |  | × |  |  |  |  |
| Ethyl acetate | Ester | C_4_H_8_O_2_ | 2.7798 | 611.6 | 730 |  |  |  |  |  |  |  |  | × |  |
| unknown |  |  | 3.0102 | 632.2 |  | × |  |  |  |  |  |  |  |  |  |
| unknown |  |  | 3.2950 | 657.6 |  |  |  |  | × |  |  |  |  |  |  |
| 1-Methoxy-2-propanol | Alcohol | C_4_H_10_O_2_ | 3.2952 | 657.6 | 857 | × |  |  | × | × |  | × |  |  |  |
| unknown |  |  | 3.3908 | 666.1 |  |  |  |  |  |  |  |  |  | × |  |
| unknown |  |  | 3.6983 | 693.6 |  |  |  |  |  |  |  | × | × |  |  |
| Pyridine | Pyridine | C_5_H_5_N | 4.1140 | 730.7 | 916 |  |  |  | × | × |  |  |  |  |  |
| unknown |  |  | 4.1189 | 731.2 |  | × | × |  |  |  |  |  |  |  |  |
| unknown |  |  | 4.6317 | 776.9 |  | × | × |  |  |  |  |  |  |  |  |
| unknown |  |  | 5.4195 | 832.5 |  | × | × |  |  |  |  |  |  |  |  |
| unknown |  |  | 5.6408 | 846.1 |  | × | × |  |  |  |  |  |  |  |  |
| unknown |  |  | 6.2306 | 882.2 |  | × | × |  |  |  |  |  |  |  |  |
| unknown |  |  | 6.3599 | 890.2 |  |  |  |  |  |  |  | × |  |  |  |
| 1-Heptanal | Aldehyde | C_7_H_14_O | 6.5595 | 902.4 | 654 |  |  |  |  |  | × |  |  |  | (Wood and Szewczak, 2007; Shi et al., 2010; Cansu et al., 2013; Li et al., 2013) |
| Hexanoic acid methyl ester | Ester | C_7_H_14_O_2_ | 6.9078 | 923.1 | 829 |  |  | × |  |  |  |  |  |  |  |
| 4-Methylnonane | Alkane | C_10_H_22_ | 7.6139 | 965.1 | 798 |  | × |  |  |  |  |  |  |  |  |
| unknown |  |  | 7.6288 | 966.0 |  | × | × |  | × |  |  |  | × |  |  |
| unknown |  |  | 7.7299 | 972.0 |  |  |  |  |  |  |  | × | × |  |  |
| 4-Methyl-3-pentenoic acid | Acid | C_6_H_10_O_2_ | 7.8892 | 981.5 | 642 |  |  |  |  |  |  | × |  |  |  |
| unknown |  |  | 7.8926 | 981.7 |  |  |  |  |  |  |  |  | × |  |  |
| unknown |  |  | 8.0599 | 991.7 |  |  |  | × |  |  |  |  |  |  |  |
| 1,3-Dichlorobenzene | Benzene | C_6_H_4_Cl_2_ | 8.3774 | 1010.9 | 907 | × | × |  | × | × |  |  |  |  |  |
| unknown |  |  | 8.5414 | 1020.9 |  |  |  |  |  |  | × |  |  |  |  |
| 2-Ethylhexanol | Alcohol | C_8_H_18_O | 8.6534 | 1027.8 | 831 |  |  |  | × | × |  | × | × |  | (Kivcak et al., 2007; Pandey and Banerjee, 2014) |
| unknown |  |  | 8.9926 | 1048.6 |  |  |  |  | × | × |  |  |  |  |  |
| unknown |  |  | 9.1305 | 1057.1 |  |  |  | × |  |  |  |  |  |  |  |
| unknown |  |  | 9.2584 | 1064.9 |  | × | × |  | × | × |  |  |  |  |  |
| 1-Octanol | Alcohol | C_8_H_18_O | 9.3361 | 1069.7 | 769 |  | × |  |  |  |  |  |  |  |  |
| unknown |  |  | 9.3400 | 1069.9 |  |  |  |  |  |  |  | × | × |  |  |
| Acetophenone | Ketone | C_8_H_8_O | 9.3447 | 1070.2 | 763 | × |  |  | × |  |  |  |  |  | (Rajabi et al., 2005; Sivakumar et al., 2008) |
| unknown |  |  | 9.6690 | 1090.1 |  |  |  |  |  |  | × |  |  |  |  |
| (E)-5-Methyl-4-decene | Alkene | C_11_H_22_ | 9.9004 | 1104.6 | 651 |  | × |  |  |  |  |  |  |  |  |
| 1-Nonanal | Aldehyde | C_9_H_18_O | 9.9030 | 1104.8 | 739 | × |  |  |  |  |  | × |  |  | (Kivcak et al., 2007; Wood and Szewczak, 2007; Pavithra et al., 2009; Hussain et al., 2017) |
| unknown |  |  | 9.9113 | 1105.3 |  |  |  |  |  |  |  |  | × |  |  |
| unknown |  |  | 10.1685 | 1122.1 |  | × | × |  |  |  |  |  |  |  |  |
| unknown |  |  | 10.2799 | 1129.4 |  | × | × |  |  | × |  |  | × |  |  |
| unknown |  |  | 10.5095 | 1144.4 |  |  |  | × |  |  |  |  |  |  |  |
| unknown |  |  | 10.6615 | 1154.3 |  |  |  | × |  |  |  |  |  |  |  |
| unknown |  |  | 10.8792 | 1168.6 |  |  |  | × |  |  |  |  |  |  |  |
| unknown |  |  | 10.9124 | 1170.7 |  |  | × |  |  | × |  | × |  |  |  |
| unknown |  |  | 11.0818 | 1181.8 |  |  |  |  | × | × |  | × | × |  |  |
| Levomenthol | Alcohol | C_10_H_20_O | 11.0999 | 1183.0 | 794 |  | × |  |  |  |  |  |  |  | (Kaya and Duran, 2018; Gharaibeh et al., 2020; Montenegro et al., 2020; Zhu et al., 2020) |
| unknown |  |  | 11.2267 | 1191.3 |  |  | × |  |  |  |  |  | × |  |  |
| 2-Decanone | Ketone | C_10_H_20_O | 11.2302 | 1191.5 | 751 |  |  |  | × |  |  | × |  |  | (Guleria et al., 2013; Zheng et al., 2013; Jayakumar et al., 2020) |
| unknown |  |  | 11.2704 | 1194.1 |  |  |  |  |  | × |  |  |  |  |  |
| 3,5-Dimethylundecane | Acyl | C_13_H_28_ | 11.3576 | 1199.8 | 610 |  | × |  |  |  |  |  |  |  |  |
| 4,6-Dimethylundecane | Acyl | C_13_H_28_ | 11.3614 | 1200.1 | 720 |  |  |  |  |  |  |  | × |  |  |
| Dodecane | Alkane | C_12_H_26_ | 11.3618 | 1200.1 | 630 |  |  |  |  |  |  | × |  |  | (Pavithra et al., 2009; Okla et al., 2019; Jayakumar et al., 2020) |
| unknown |  |  | 11.3681 | 1200.6 |  | × |  | × | × |  |  |  |  |  |  |
| 1-Decanal | Aldehyde | C_10_H_20_O | 11.4522 | 1206.4 | 824 | × | × |  |  |  |  |  |  |  | (Liu et al., 2012; Kazemi and Sharifi, 2017) |
| unknown |  |  | 11.5178 | 1210.9 |  | × |  |  | × | × |  |  | × |  |  |
| unknown |  |  | 11.6050 | 1216.9 |  |  | × |  | × |  |  | × |  |  |  |
| unknown |  |  | 11.6902 | 1222.8 |  | × |  |  |  |  |  |  |  |  |  |
| unknown |  |  | 11.7579 | 1227.4 |  |  | × |  |  |  |  |  |  |  |  |
| 2,4-Dimethylacetophenone | Phenone | C_10_H_12_O | 11.7644 | 1227.9 | 697 |  |  |  |  |  |  |  | × |  |  |
| unknown |  |  | 11.8209 | 1231.8 |  |  |  |  |  |  | × | × |  | × |  |
| 1,3-Benzothiazole | Benzothia-zole | C_7_H_5_NS | 11.8901 | 1236.6 | 923 | × | × |  | × |  |  |  |  |  | (Bondock et al., 2010; Shi et al., 2010; Li et al., 2012) |
| unknown |  |  | 11.9854 | 1243.1 |  | × | × | × | × | × |  |  | × |  |  |
| unknown |  |  | 12.1096 | 1251.7 |  | × | × | × | × |  |  | × | × |  |  |
| 1,3-Di-tert-butylbenzene | Benzene | C_14_H_22_ | 12.1156 | 1252.1 | 795 |  |  |  |  |  | × |  |  |  |  |
| unknown |  |  | 12.2002 | 1257.9 |  | × | × | × |  |  |  | × | × |  |  |
| unknown |  |  | 12.4475 | 1275.0 |  | × |  |  |  |  |  | × |  |  |  |
| 2,6,11-Trimethyldodecane | Alkane | C_15_H_32_ | 12.4483 | 1275.1 | 859 |  | × |  |  | × |  |  | × |  |  |
| unknown |  |  | 12.4535 | 1275.4 |  |  |  |  | × |  |  | × | × |  |  |
| unknown |  |  | 12.5696 | 1283.4 |  | × |  |  |  |  |  | × | × |  |  |
| 2-Butyl-1-octanol | Alcohol | C_12_H_26_O | 12.5708 | 1283.5 | 727 |  | × |  |  |  |  |  |  |  |  |
| 1-Undecanol | Alcohol | C_11_H_24_O | 12.5800 | 1284.1 | 640 |  |  |  |  |  |  |  | × |  | (Togashi et al., 2007) |
| 1-Dodecene | Alkene | C_12_H_24_ | 12.5803 | 1284.2 | 660 |  |  |  |  |  |  | × |  |  | (Roy et al., 2009; Abdelwahab et al., 2010) |
| unknown |  |  | 12.7172 | 1293.6 |  | × | × | × | × | × |  | × | × |  |  |
| 1-Tridecene | Alkene | C_13_H_26_ | 12.8175 | 1300.5 | 788 | × | × |  | × | × |  | × |  |  | (Kumar et al., 2011; Satmi and Hossain, 2016) |
| unknown |  |  | 12.8186 | 1300.6 |  |  |  | × |  |  |  | × |  |  |  |
| unknown |  |  | 12.9390 | 1308.9 |  | × | × | × |  | × | × | × | × |  |  |
| 2,2,4,4,6,8,8-Heptamethylnonane | Alkane | C_16_H_34_ | 13.0251 | 1314.8 | 635 |  | × |  |  |  |  |  |  |  |  |
| unknown |  |  | 13.0507 | 1316.6 |  | × |  |  |  |  |  | × |  |  |  |
| unknown |  |  | 13.2103 | 1327.6 |  | × | × |  | × |  | × | × |  |  |  |
| 4,6-Dimethyldodecane | Alkane | C_14_H_30_ | 13.2312 | 1329.1 | 852 |  | × |  |  |  |  |  | × |  |  |
| unknown |  |  | 13.3473 | 1337.1 |  | × | × |  |  | × |  | × |  |  |  |
| 6-Ethyltetralin | Naphtalene | C_12_H_16_ | 13.3963 | 1340.4 | 684 |  |  |  | × |  |  |  |  |  |  |
| unknown |  |  | 13.4385 | 1343.3 |  |  |  | × |  |  |  | × |  |  |  |
| 5-Methyltridecane | Alkane | C_14_H_30_ | 13.4835 | 1346.5 | 710 |  |  |  |  |  |  |  | × |  |  |
| Oxymethylencampher | Ketone | C_11_H_16_O_2_ | 13.4903 | 1346.9 | 637 |  |  |  |  |  |  | × | × | × |  |
| unknown |  |  | 13.5701 | 1352.4 |  | × | × |  |  |  |  | × | × |  |  |
| unknown |  |  | 13.6573 | 1358.4 |  |  |  | × |  |  |  | × | × |  |  |
| 2-Tert-butyl-4-methylphenol | Phenol | C_11_H_16_O | 13.6620 | 1358.8 | 744 |  |  |  | × |  |  |  | × |  |  |
| unknown |  |  | 13.7703 | 1366.2 |  |  | × |  |  |  |  |  |  |  |  |
| 2-Ethyl-3-hydroxyhexyl  2-methylpropanoate | Ester | C_12_H_24_O_3_ | 13.8539 | 1372.0 | 666 | × |  |  |  |  |  |  |  |  | (Li et al., 2012) |
| unknown |  |  | 13.8713 | 1373.2 |  |  | × | × |  |  |  | × |  |  |  |
| 1-Tetradecene | Alkene | C_14_H_28_ | 14.0589 | 1386.1 | 683 |  |  | × |  |  |  |  |  |  | (Palic et al., 2002; Tayung et al., 2011) |
| unknown |  |  | 14.1905 | 1395.2 |  |  |  |  |  |  |  | × |  |  |  |
| 1-Dodecanal | Aldehyde | C_12_H_24_O | 14.2952 | 1402.4 | 674 |  |  |  |  |  |  | × |  |  | (Kubo et al., 2004; Dordevic et al., 2011; Boussalah, 2020) |
| unknown |  |  | 14.3209 | 1404.2 |  |  |  | × | × | × |  |  |  |  |  |
| unknown |  |  | 14.4879 | 1415.7 |  |  | × |  |  |  |  |  |  | × |  |
| unknown |  |  | 14.7102 | 1431.0 |  |  |  |  |  |  |  | × | × |  |  |
| Nerylacetone | Ketone | C_13_H_22_O | 14.7857 | 1463.3 | 788 | × | × |  |  |  |  |  | × |  | (Zellagui et al., 2012; Kazemi and Sharifi, 2017) |
| unknown |  |  | 14.8145 | 1438.2 |  |  |  |  |  |  |  | × | × |  |  |
| Dimethylphthalate | Benzene | C_10_H_10_O_4_ | 14.8155 | 1438.3 | 711 |  |  |  |  |  |  | × |  |  |  |
| unknown |  |  | 14.9084 | 1444.7 |  |  | × | × | × | × | × | × | × |  |  |
| unknown |  |  | 15.0489 | 1454.4 |  | × | × |  |  | × |  | × | × |  |  |
| 1-Dodecanol | Alcohol | C_12_H_26_O | 15.1110 | 1458.7 | 809 |  | × | × | × |  |  | × |  |  | (Togashi et al., 2007; Vairappan et al., 2012) |
| unknown |  |  | 15.1796 | 1463.4 |  | × | × |  |  |  |  |  | × |  |  |
| unknown |  |  | 15.2999 | 1471.7 |  | × | × |  |  |  |  |  |  |  |  |
| unknown |  |  | 15.3972 | 1478.4 |  | × | × |  | × | × |  |  | × |  |  |
| unknown |  |  | 15.4876 | 1484.7 |  | × | × | × |  |  |  | × | × |  |  |
| unknown |  |  | 15.6492 | 1495.8 |  |  |  | × |  |  |  |  |  |  |  |
| unknown |  |  | 15.7408 | 1502.1 |  | × | × | × |  |  |  |  |  |  |  |
| Pentadecane | Alkane | C_15_H_32_ | 15.8593 | 1510.3 | 791 |  |  |  |  |  |  | × | × |  | (Ozdemir et al., 2004; Hussain et al., 2017) |
| 5-Propyltridecane | Alkane | C_16_H_34_ | 15.8602 | 1510.4 | 788 |  | × |  |  |  |  |  |  |  |  |
| unknown |  |  | 15.8608 | 1510.4 |  | × |  |  |  |  |  | × |  |  |  |
| unknown |  |  | 15.9824 | 1518.8 |  | × |  | × |  |  |  | × | × |  |  |
| unknown |  |  | 16.0950 | 1526.6 |  | × | × |  |  |  |  | × |  |  |  |
| 1,1,4,5,6-Pentamethylindane | Indane | C_14_H_20_ | 16.1394 | 1529.6 | 673 |  |  | × |  |  |  |  |  |  |  |
| unknown |  |  | 16.1994 | 1533.7 |  | × | × |  |  |  |  |  |  |  |  |
| unknown |  |  | 16.3143 | 1541.7 |  |  |  |  |  |  |  | × |  |  |  |
| unknown |  |  | 16.4817 | 1553.2 |  | × | × |  |  |  |  | × | × |  |  |
| unknown |  |  | 16.5991 | 1561.3 |  |  |  | × |  |  |  | × | × |  |  |
| unknown |  |  | 16.6885 | 1567.5 |  | × | × |  |  |  |  |  |  |  |  |
| unknown |  |  | 16.8103 | 1575.9 |  | × | × |  |  |  |  |  |  |  |  |
| unknown |  |  | 16.9303 | 1584.2 |  | × | × |  | × |  |  | × | × |  |  |
| unknown |  |  | 17.1503 | 1599.3 |  | × | × | × |  |  |  |  | × |  |  |
| unknown |  |  | 17.2406 | 1605.6 |  | × | × |  | × |  |  | × |  |  |  |
| unknown |  |  | 17.3376 | 1612.2 |  |  | × |  |  | × |  | × | × |  |  |
| unknown |  |  | 17.4680 | 1621.2 |  | × | × |  | × |  |  | × | × |  |  |
| unknown |  |  | 17.6339 | 1633.1 |  | × | × |  | × |  |  |  | × |  |  |
| unknown |  |  | 17.7798 | 1642.7 |  | × | × |  |  |  |  |  | × |  |  |
| unknown |  |  | 17.9592 | 1655.1 |  |  | × |  | × |  | × |  |  |  |  |
| unknown |  |  | 18.0542 | 1661.7 |  |  |  |  | × |  | × |  |  |  |  |
| 1,3-Diisopropylnaphthalene | Naphtalene | C_16_H_20_ | 18.0794 | 1663.4 | 713 | × | × |  |  |  |  | × | × |  |  |
| unknown |  |  | 18.1591 | 1668.9 |  |  | × |  |  |  |  | × |  |  |  |
| 1,7-Diisopropylnaphthalene | Naphtalene | C_16_H_20_ | 18.1612 | 1669.1 | 708 | × |  |  |  |  |  |  |  |  |  |
| unknown |  |  | 18.2786 | 1677.1 |  |  |  |  |  |  | × |  |  |  |  |
| Propyl laurate | Ester | C_15_H_30_O_2_ | 18.3904 | 1684.9 | 607 | × |  |  |  |  |  |  |  |  |  |
| unknown |  |  | 18.3933 | 1685.1 |  | × | × |  |  |  |  |  | × |  |  |
| unknown |  |  | 18.4958 | 1692.1 |  | × | × |  |  |  |  | × |  |  |  |
| unknown |  |  | 18.6300 | 1701.4 |  |  |  |  | × |  |  | × | × |  |  |
| unknown |  |  | 18.8801 | 1718.6 |  |  | × |  |  |  |  |  |  |  |  |
| unknown |  |  | 19.0014 | 1727.0 |  | × |  |  |  |  |  | × | × |  |  |
| Isobutyl 2-isobutoxybenzoate | Benzene | C_15_H_22_O_3_ | 19.0029 | 1727.1 | 755 |  | × |  |  |  |  |  |  |  |  |
| Myristic acid methyl ester | Acid | C_15_H_30_O_2_ | 19.0829 | 1732.6 | 640 |  |  |  |  |  |  |  | × |  |  |
| unknown |  |  | 19.1177 | 1735.0 |  | × | × |  | × | × |  |  |  |  |  |
| unknown |  |  | 19.2503 | 1744.2 |  |  |  |  | × |  |  |  | × |  |  |
| Myristic acid | Acid | C_14_H_28_O_2_ | 19.4584 | 1758.5 | 706 | × |  |  |  |  |  | × |  |  | (Agoramoorthy et al., 2007; Altieri et al., 2009; Chen et al., 2019) |
| unknown |  |  | 19.4612 | 1758.7 |  |  | × |  |  |  |  | × | × |  |  |
| unknown |  |  | 19.5693 | 1772.4 |  | × |  |  |  |  |  |  |  |  |  |
| unknown |  |  | 19.5694 | 1766.2 |  | × | × |  |  |  |  |  |  |  |  |
| unknown |  |  | 19.8204 | 1783.5 |  | × | × |  |  |  |  |  |  |  |  |
| unknown |  |  | 19.9582 | 1793.0 |  | × | × |  | × |  |  | × | × |  |  |
| unknown |  |  | 20.0821 | 1801.5 |  |  |  |  |  |  |  | × | × |  |  |
| unknown |  |  | 20.1995 | 1809.6 |  | × | × |  |  |  |  | × |  |  |  |
| unknown |  |  | 20.3203 | 1818.0 |  |  |  |  |  | × |  |  | × |  |  |
| unknown |  |  | 20.4102 | 1824.2 |  | × | × |  | × | × | × | × | × |  |  |
| unknown |  |  | 20.6290 | 1839.2 |  | × | × |  |  |  |  | × | × |  |  |
| unknown |  |  | 20.9175 | 1859.1 |  | × | × |  | × |  |  |  |  |  |  |
| unknown |  |  | 21.1084 | 1872.3 |  | × | × |  |  |  |  |  |  |  |  |
| unknown |  |  | 21.5389 | 1902.0 |  | × | × |  |  |  |  | × | × |  |  |
| unknown |  |  | 22.4444 | 1964.4 |  |  |  |  | × |  |  |  |  |  |  |
| unknown |  |  | 22.7400 | 1984.8 |  | × | × |  | × | × |  | × | × |  |  |
| unknown |  |  | 22.8425 | 1991.9 |  |  | × |  |  |  |  |  |  |  |  |
| unknown |  |  | 22.9389 | 1998.5 |  |  |  |  |  |  | × |  |  |  |  |
| unknown |  |  | 23.1899 | 2015.9 |  |  | × |  |  |  | × | × | × |  |  |
| unknown |  |  | 23.6183 | 2045.4 |  | × | × |  |  |  |  |  | × |  |  |
| unknown |  |  | 23.7388 | 2053.7 |  | × | × |  |  |  |  |  |  |  |  |
| unknown |  |  | 23.8297 | 2060.0 |  |  |  |  |  |  |  |  | × |  |  |
| Heneicosane | Alkane | C_21_H_44_ | 24.4592 | 2103.4 | 839 |  |  |  |  |  |  | × | × |  | (Boussaada et al., 2008; Kotan et al., 2010) |
| unknown |  |  | 24.4600 | 2103.4 |  |  | × |  |  |  |  |  |  |  |  |
| unknown |  |  | 24.6040 | 2113.4 |  | × | × |  |  |  |  |  |  |  |  |
| unknown |  |  | 25.0016 | 2140.8 |  |  |  |  |  |  | × |  |  |  |  |
| unknown |  |  | 25.2496 | 2157.9 |  | × | × |  | × |  |  | × | × |  |  |
| unknown |  |  | 25.4398 | 2171.0 |  | × | × |  |  |  |  |  |  |  |  |
| unknown |  |  | 25.7011 | 2189.0 |  |  |  | × |  |  |  |  |  |  |  |
| unknown |  |  | 25.8890 | 2202.0 |  | × | × |  |  |  |  |  |  |  |  |
| Docosane | Alkane | C_22_H_46_ | 26.0210 | 2211.1 | 728 |  |  |  |  |  |  | × | × |  | (Sinek et al., 2012; Wang et al., 2015) |
| unknown |  |  | 26.0337 | 2212.0 |  |  |  |  | × |  |  |  |  |  |  |
| unknown |  |  | 26.2400 | 2226.2 |  |  |  | × |  |  | × |  |  |  |  |
| unknown |  |  | 26.3792 | 2235.8 |  |  |  | × |  |  |  |  |  |  |  |
| unknown |  |  | 26.6111 | 2251.8 |  |  |  | × |  |  |  |  |  |  |  |
| unknown |  |  | 26.7598 | 2262.1 |  | × | × |  |  |  |  | × |  |  |  |
| Dodecyl 2-pentyl ester  sulfurous acid | Sulfurous acid | C_17_H_36_O_3_S | 26.7698 | 2262.7 | 606 |  |  |  |  |  |  |  | × |  |  |
| unknown |  |  | 26.8479 | 2268.1 |  |  |  |  |  |  | × |  |  |  |  |
| unknown |  |  | 27.1090 | 2286.1 |  |  |  |  |  |  | × | × | × |  |  |
| unknown |  |  | 27.2989 | 2299.2 |  | × | × |  |  |  |  |  |  |  |  |
| unknown |  |  | 27.4880 | 2312.3 |  | × | × |  |  |  |  |  |  |  |  |
| Methyl copalate | Terpenoid | C_21_H_34_O_2_ | 27.5995 | 2320.0 | 652 | × |  |  |  |  |  |  |  |  |  |
| unknown |  |  | 27.6006 | 2320.0 |  |  | × | × | × |  |  | × | × |  |  |
| unknown |  |  | 27.8286 | 2335.8 |  |  |  | × |  |  |  |  |  |  |  |
| unknown |  |  | 28.0098 | 2348.3 |  |  |  | × |  |  |  |  |  |  |  |
| 2-Methyltricosane | Alkane | C_24_H_50_ | 28.1795 | 2360.0 | 755 |  | × |  |  |  |  |  |  |  |  |
| unknown |  |  | 28.1800 | 2360.0 |  | × |  |  |  |  |  |  |  |  |  |
| unknown |  |  | 28.3610 | 2372.5 |  |  |  |  |  | × | × |  |  |  |  |
| unknown |  |  | 28.4867 | 2381.1 |  |  |  |  |  |  |  | × | × |  |  |
| unknown |  |  | 28.6392 | 2391.7 |  |  |  |  |  |  | × |  |  |  |  |
| unknown |  |  | 28.9019 | 2409.8 |  |  |  | × |  |  |  |  |  |  |  |
| unknown |  |  | 29.0184 | 2417.8 |  |  |  |  |  |  | × |  |  |  |  |
| unknown |  |  | 29.4089 | 2444.8 |  |  |  | × |  |  |  |  |  |  |  |
| unknown |  |  | 29.5609 | 2455.2 |  |  |  |  |  |  | × |  |  |  |  |
| unknown |  |  | 29.8282 | 2473.7 |  | × | × |  |  |  |  |  |  | × |  |
| unknown |  |  | 29.9189 | 2479.9 |  |  |  | × |  |  |  |  |  |  |  |
| unknown |  |  | 29.9206 | 2480.0 |  |  |  |  |  |  | × |  |  |  |  |
|  |  |  |  |  |  |  |  |  |  |  |  |  |  |  |  |

# References

Abdelwahab, S. I., Zaman, F. Q., Mariod, A. A., Yaacob, M., Abdelmageed, A. H. A., and Khamis, S. (2010). Chemical composition, antioxidant and antibacterial properties of the essential oils of Etlingera elatior and Cinnamomum pubescens Kochummen. *J. Sci. Food Agric.* 90, 2682–2688. doi:10.1002/jsfa.4140.

Agoramoorthy, G., Chandrasekaran, M., Venkatesalu, V., and Hsu, M. J. (2007). Antibacterial and antifungal activities of fatty acid methyl esters of the blind-your-eye mangrove from India. *Braz. J. Microbiol.* 38, 739–742.

Altieri, C., Bevilacqua, A., Cardillo, D., and Sinigaglia, M. (2009). Antifungal activity of fatty acids and their monoglycerides against Fusarium spp. in a laboratory medium. *Int. J. Food Sci. Technol.* 44, 242–245. doi:10.1111/j.1365-2621.2007.01639.x.

Bondock, S., Fadaly, W., and Metwally, M. A. (2010). Synthesis and antimicrobial activity of some new thiazole, thiophene and pyrazole derivatives containing benzothiazole moiety. *Eur. J. Med. Chem.* 45, 3692–3701. doi:10.1016/j.ejmech.2010.05.018.

Boussaada, O., Saidana, D., Chriaa, J., Chraif, I., Ammar, Mahjoub, M. A., et al. (2008). Chemical composition and antimicrobial activity of volatile components of Scorzonera undulata. *J. Essent. Oil Res.* 20, 358–362. doi:10.1080/10412905.2008.9700030.

Boussalah, N. (2020). Chemical Composition and Biological Activities of Essential Oil and Hydrosol Extract from Aerial Parts of Cynoglossum cheirifolium L. from Algeria. *J. Essent. Oil Bear. Plants* 23, 97–104. doi:10.1080/0972060X.2020.1729249.

Cansu, T. B., Yayli, B., Ozdemir, T., Batan, N., Alpay Karaoglu, S., and Yayli, N. (2013). Antimicrobial activity and chemical composition of the essential oils of mosses (Hylocomium splendens (Hedw.) Schimp. and Leucodon sciuroides (Hedw.) Schwagr.) growing in Turkey. *Turk. J. Chem.* 37, 213–219. doi:10.3906/kim-1204-72.

Chen, X., Zhao, X., Deng, Y., Bu, X., Ye, H., and Guo, N. (2019). Antimicrobial potential of myristic acid against Listeria monocytogenes in milk. *J. Antibiot. (Tokyo)* 72, 298–305. doi:10.1038/s41429-019-0152-5.

Dordevic, A., Zlatkovic, B., Lazarevic, J., and Palic, R. (2011). A detailed chemical composition and antimicrobial activity of Hypericum richeri Vill. subsp grisebachii (Boiss.) Nyman essential oil from Serbia. *J. Med. Plants Res.* 5, 5486–5492.

Gharaibeh, M. H., Khalifeh, M. S., Zattout, E. M., and Abu-Qatouseh, L. F. (2020). Potential antimicrobial effect of plant essential oils and virulence genes expression in methicillin-resistant Staphylococcus aureus isolates. *Vet. World* 13, 669–675. doi:10.14202/vetworld.2020.669-675.

Guleria, S., Tiku, A. K., Koul, A., Gupta, S., Singh, G., and Razdan, V. K. (2013). Antioxidant and Antimicrobial Properties of the Essential Oil and Extracts of Zanthoxylum alatum Grown in North-Western Himalaya. *Sci. World J.* 2013. doi:10.1155/2013/790580.

Hussain, A., Tian, M.-Y., and Wen, S.-Y. (2017). Exploring the Caste-Specific Multi-Layer Defense Mechanism of Formosan Subterranean Termites, Coptotermes formosanus Shiraki. *Int. J. Mol. Sci.* 18, 2694. doi:10.3390/ijms18122694.

Jayakumar, V., Ramesh Sundar, A., and Viswanathan, R. (2020). Biocontrol of Colletotrichum falcatum with volatile metabolites produced by endophytic bacteria and profiling VOCs by headspace SPME coupled with GC–MS. *Sugar Tech* 23, 94–107. doi:10.1007/s12355-020-00891-2.

Kaya, D. A., and Duran, N. (2018). *The antimicrobial activities of Myrtus communis and Micromeria fruticosa essential oils*. Bucharest: Incdtp-Icpi doi:10.24264/icams-2018.IV.2.

Kazemi, M., and Sharifi, M. (2017). Composition, Antimicrobial and Antioxidant Activities of Essential Oil of Stachys kermanshahensis. *Chem. Nat. Compd.* 53, 767–769. doi:10.1007/s10600-017-2116-y.

Kivcak, B., Mert, T., Saglam, H., Ozturk, T., Kurkcuoglu, M., and Baser, K. H. C. (2007). Chemical composition and antimicrobial activity of the essential oil of Anthemis wiedemanniana from Turkey. *Chem. Nat. Compd.* 43, 47–51. doi:10.1007/s10600-007-0029-x.

Kotan, R., Cakir, A., Dadasoglu, F., Aydin, T., Cakmakci, R., Ozer, H., et al. (2010). Antibacterial activities of essential oils and extracts of Turkish Achillea, Satureja and Thymus species against plant pathogenic bacteria. *J. Sci. Food Agric.* 90, 145–160. doi:10.1002/jsfa.3799.

Kubo, I., Fujita, K., Kubo, A., Nihei, K., and Ogura, T. (2004). Antibacterial Activity of Coriander Volatile Compounds against Salmonella choleraesuis. *J. Agric. Food Chem.* 52, 3329–3332. doi:10.1021/jf0354186.

Kumar, V., Bhatnagar, A. K., and Srivastava, J. N. (2011). Antibacterial activity of crude extracts of Spirulina platensisand its structural elucidation of bioactive compound. *J. Med. Plants Res.* 5, 7043–7048. doi:10.5897/JMPR11.1175.

Li, M., Han, G., Chen, H., Yu, J., and Zhang, Y. (2012). Chemical compounds and antimicrobial activity of volatile oils from bast and fibers of Apocynum venetum. *Fibers Polym.* 13, 322–328. doi:10.1007/s12221-012-0322-6.

Li, Z.-J., Njateng, G. S. S., He, W.-J., Zhang, H.-X., Gu, J.-L., Chen, S.-N., et al. (2013). Chemical Composition and Antimicrobial Activity of the Essential Oil from the Edible Aromatic Plant Aristolochia delavayi. *Chem. Biodivers.* 10, 2032–2041. doi:10.1002/cbdv.201300066.

Liu, K., Chen, Q., Liu, Y., Zhou, X., and Wang, X. (2012). Isolation and Biological Activities of Decanal, Linalool, Valencene, and Octanal from Sweet Orange Oil. *J. Food Sci.* 77, C1156–C1161. doi:10.1111/j.1750-3841.2012.02924.x.

Montenegro, I., Said, B., Godoy, P., Besoain, X., Parra, C., Diaz, K., et al. (2020). Antifungal Activity of Essential Oil and Main Components from Mentha pulegium Growing Wild on the Chilean Central Coast. *Agron.-Basel* 10, 254. doi:10.3390/agronomy10020254.

Okla, M. K., Alamri, S. A., Salem, M. Z. M., Ali, H. M., Behiry, S., Nasser, R. A., et al. (2019). Yield, Phytochemical Constituents, and Antibacterial Activity of Essential Oils from the Leaves/Twigs, Branches, Branch Wood, and Branch Bark of Sour Orange (Citrus aurantium L.). *Processes* 7, 363. doi:10.3390/pr7060363.

Ozdemir, G., Karabay, N. U., Dalay, M. C., and Pazarbasi, B. (2004). Antibacterial activity of volatile component and various extracts of Spirulina platensis. *Phytother. Res.* 18, 754–757. doi:10.1002/ptr.1541.

Palic, R., Stojanovic, G., Alagic, S., Nikolic, M., and Lepojevic, Z. (2002). Chemical composition and antimicrobial activity of the essential oil and CO2 extracts of the oriental tobacco, Prilep. *Flavour Fragr. J.* 17, 323–326. doi:10.1002/ffj.1084.

Pandey, A., and Banerjee, D. (2014). Daldinia bambusicola Ch4/11 an Endophytic Fungus Producing Volatile Organic Compounds Having Antimicrobial and Olio Chemical Potential. *J. Adv. Microbiol.* 6, 330–337.

Pavithra, P. S., Sreevidya, N., and Verma, R. S. (2009). Antibacterial activity and chemical composition of essential oil of Pamburus missionis. *J. Ethnopharmacol.* 124, 151–153. doi:10.1016/j.jep.2009.04.016.

Rajabi, L., Courreges, C., Montoya, J., Aguilera, R. J., and Primm, T. P. (2005). Acetophenones with selective antimycobacterial activity. *Lett. Appl. Microbiol.* 40, 212–217. doi:10.1111/j.1472-765X.2005.01657.x.

Roy, S., Rao, K., Bhuvaneswari, Ch., Giri, A., and Mangamoori, L. N. (2009). Phytochemical analysis of Andrographis paniculata extract and its antimicrobial activity. *World J. Microbiol. Biotechnol.* 26, 85. doi:10.1007/s11274-009-0146-8.

Satmi, F. R. S., and Hossain, M. A. (2016). In vitro antimicrobial potential of crude extracts and chemical compositions of essential oils of leaves of Mentha piperita L native to the Sultanate of Oman. *Pac. Sci. Rev. Nat. Sci. Eng.* 18, 103–106. doi:10.1016/j.psra.2016.09.005.

Shi, B., Liu, W., Wei, S., and Wu, W. (2010). Chemical Composition, Antibacterial and Antioxidant Activity of the Essential Oil of Bupleurum longiradiatum. *Nat. Prod. Commun.* 5, 1139–1142. doi:10.1177/1934578X1000500734.

Sinek, K., Iskender, N. Y., Yayli, B., Karaoglu, S. A., and Yayli, N. (2012). Antimicrobial Activity and Chemical Composition of the Essential Oil from Campanula glomerata L. Subsp Hispida (Witasek) Hayek. *Asian J. Chem.* 24, 1931–1934.

Sivakumar, P. M., Sheshayan, G., and Doble, M. (2008). Experimental and QSAR of Acetophenones as Antibacterial Agents. *Chem. Biol. Drug Des.* 72, 303–313. doi:10.1111/j.1747-0285.2008.00702.x.

Tayung, K., Barik, B., Jha, D., and Deka, D. (2011). Identification and characterization of antimicrobial metabolite from an endophytic fungus, Fusariumsolani isolated from bark of Himalayan yew. *Mycosphere* 2, 203–213.

Togashi, N., Shiraishi, A., Nishizaka, M., Matsuoka, K., Endo, K., Hamashima, H., et al. (2007). Antibacterial activity of long-chain fatty alcohols against Staphylococcus aureus. *Molecules* 12, 139–148. doi:10.3390/12020139.

Vairappan, C. S., Nagappan, T., and Palaniveloo, K. (2012). Essential Oil Composition, Cytotoxic and Antibacterial Activities of Five Etlingera Species from Borneo. *Nat. Prod. Commun.* 7, 239–249. doi:10.1177/1934578X1200700233.

Wang, L., Elliott, B., Jin, X., Zeng, L., and Chen, J. (2015). Antimicrobial properties of nest volatiles in red imported fire ants, Solenopsis invicta (hymenoptera: formicidae). *Sci. Nat.* 102, 66. doi:10.1007/s00114-015-1316-1.

Wood, W. F., and Szewczak, J. M. (2007). Volatile antimicrobial compounds in the pelage of the Mexican free-tailed bat, Tadarida brasiliensis mexicana. *Biochem. Syst. Ecol.* 35, 566–568. doi:10.1016/j.bse.2007.04.002.

Zellagui, A., Gherraf, N., and Rhouati, S. (2012). Chemical composition and antibacterial activity of the essential oils of Ferula vesceritensis Coss et Dur. leaves, endemic in Algeria. *Org. Med. Chem. Lett.* 2, 31. doi:10.1186/2191-2858-2-31.

Zheng, M., Shi, J., Shi, J., Wang, Q., and Li, Y. (2013). Antimicrobial effects of volatiles produced by two antagonistic Bacillus strains on the anthracnose pathogen in postharvest mangos. *Biol. Control* 65, 200–206. doi:10.1016/j.biocontrol.2013.02.004.

Zhu, Q., Jiang, M.-L., Shao, F., Ma, G.-Q., Shi, Q., and Liu, R.-H. (2020). Chemical Composition and Antimicrobial Activity of the Essential Oil From Euphorbia helioscopia L. *Nat. Prod. Commun.* 15, 1–6. doi:10.1177/1934578X20953249.
